# Seed morphology and germination of <*Turnera diffusa*> Willd. ex Schult. emulating environmental conditions within ant nest

**DOI:** 10.1371/journal.pone.0292626

**Published:** 2023-10-20

**Authors:** Paola Scarlet Puga-Guzmán, Fabiola Magallán-Hernández, Mónica Queijeiro-Bolaños, Juan Antonio Valencia-Hernández, Santiago Vergara-Pineda

**Affiliations:** Faculty of Natural Sciences, Environmental Horticulture Group, Autonomous University of Queretaro—Juriquilla Campus, Querétaro, Mexico; National Institute of Agricultural Research - INRA, MOROCCO

## Abstract

Damiana (*Turnera diffusa* Willd. ex Schult.) is a species of plant used in traditional Mexican medicine for its aphrodisiac properties. Although it has a high commercial demand, both nationally and internationally, its sexual propagation is not usual due to the low percentage of seed germination. It has been proposed that ants play an important role in germination, due to the presence of elaiosomes. Therefore, the objectives of this study were to characterize the seed morphology of *T*. *diffusa* for agronomic purposes, analyze their viability, and evaluate their germination by simulating environmental conditions of an ant nest. For the morphological characterization, 30 seeds were selected and evaluated for the variables of color, size, and weight. Viability was evaluated with a tetrazolium test using two lots of seeds collected in 2016 and 2017, with different concentrations and three exposure times at 40°C. The germination of *T*. *diffusa* was evaluated under three pre-germination treatments and nine germination treatments. The results of the study showed that the seeds of *T*. *diffusa* have an average size of 0.725 mm long and 0.182 mm wide; the color of the seeds varies from brown to black when ripe and yellowish white when immature. There are no significant differences in the viability percentage (60%) for seeds collected in 2016 and 2017 (p = 0.20). On the other hand, there are significant differences between all the pre-germination and germination tests analyzed. Seeds of *T*. *diffusa* have the highest percentage of germination (36%) with the presence of elaiosome and 500 ppm of GA_3_. The germination interval of the seeds occurs over a period of six to 39 days. The application of GA_3_ in the germination of the seeds indicates that they present a physiological latency which was inhibited at concentrations of 500 and 300 ppm.

## Introduction

The plant species commonly known as “damiana” (*Turnera diffusa* Willd. ex Schult.), is considered the most important species of the genus *Turnera* due to its diverse traditional uses, such as a tonic, stimulant, and aphrodisiac. It also has medicinal properties and is employed to treat colds, headaches, coughs, and gastrointestinal ailments. Other common names include “hierba de la pastora”, “hierba del venado” and “pastorcilla” [1]. Following the classification proposed by APG IV [2], the 135 species of the genus *Turnera* belong to the family Passifloraceae, although some botanists maintain that it should continue to be recognized within the separate family Turneraceae [3]. Damiana is widely distributed from California and Texas in the southwestern United States, through Mexico and Central America to Bolivia in South America. In Mexico, it preferentially grows in arid and semiarid regions. It is a shrub reaching 2 m tall with alternate to verticillate, simple leaves that measure 10 to 25 mm long. The flowers are 8 to 12 mm long, yellow, solitary, and with five petals.

Damiana has a high commercial demand globally, due to its properties as an aphrodisiac and its stimulating effect on the central nervous system. It is administered through various pharmaceutical methods such as capsules, infusions, extracts, liquors, essential oils, and cosmetic products [4, 5]. Some studies have mentioned that the raw material for these products is obtained primarily from wild populations, although the percentage or number of tons of material harvested directly from its natural habitat is not available. Given the lack of sustainable management plans, there is a progressive reduction and discontinuous production of wild populations of damiana, thus resulting in price fluctuations [5, 6].

Although damiana is currently cultivated, mostly in northern Mexico, the primary method of propagation is asexual using cuttings. Because of the importance of the species, there has been research documenting propagation using tissue cultures, however, damiana plants obtained by this technique tend to lose moisture when they are transplanted, this causes wilting and sometimes the death of the plants, which is why finally a transplant survival of 45 to 66% is obtained [7]. Regarding cultivation by seeds, various studies have shown that the species is not easily propagated by this method since seeds do not germinate under controlled conditions, and when there is germination, the percentage is very low [7–9].

Propagation by seeds is of utmost importance in maintaining the genetic diversity of crops, and this method bestows increased capacity for adaptation and resistance to pests, to mention just a few benefits. Nonetheless, to date it has not been possible to develop commercial cultivation by seeds for lack of protocols that achieve a germination percentage sufficient for mass production [7]. For this reason, research on seed germination will allow the development of conditions adequate to implement its cultivation.

Various studies have shown that the aril present on seeds of *Turnera* species forms an important link in ecological interactions with ants that influences germination. Cuautle [10] referred to the aril on the seeds as an elaiosome and analyzed the effect of seed germination of *Turnera ulmifolia* manipulated by the ant *Forelius analis*. In this context, in this research hypothesized that due to the seeds of *T*. *diffusa* possessing an elaiosome, there was likely an interaction with ants and that seeds would have an increased percentage of germination if they were subjected to environmental conditions simulating those within an ant nest. The objectives of the current research were to 1) Characterize the seed morphology of *T*. *diffusa*, 2) Determine the viability of seeds of *T*. *diffusa*, 3) Evaluate the effect of simulating the environmental conditions of an ant nest on the germination of *T*. *diffusa*, and 4) Evaluate the effect of gibberellic acid (GA_3_) on breaking seed dormancy.

## Material and methods

### Collection and storage

Seeds were collected from a wild population of *T*. *diffusa* at Maguey Verde, Peñamiller, Querétaro, México (21° 05’ 85” N, 99°41’ 77” W). The vegetation of the area is xerophytic scrub. Fruits and seeds mature and immature were collected using garden clippers, and the material was placed in labeled paper bags. In the laboratory, seeds were separated from the fruits using thin nosed tweezers and a magnifying desktop lamp (Steren HER-730BL^®^, China). The mature seeds were stored in jars with silica gel at room temperature (23°C) and in shade. The seeds for the germination experiments were collected in November 2017 and sown in May 2018.

### Characterization of seed morphology

Seed morphology was characterized with a sample of 100 seeds selected at random and by studying qualitative and quantitative features. To obtain the average size, the length and width of 30 seeds was measured using a digital caliper (Traceable^®^). A sample of 100 seeds was weighed on an analytical balance (Denver Instruments APX-200^®^, USA) and then the average weight of each seed was estimated. This procedure was necessary because of the small seed size and the inability to get a precise weight for individual seeds. The color of mature and immature seeds and fruits was determined using Munsell color charts [11]. To describe the shape and surface of the seeds, fresh fruits with mature seeds were selected and observed with a scanning electron microscope (SEM) (Carl Zeiss EVO-50^®^, Germany), using variable pressure mode. The description of the seeds was based on the terminology of Moreno [12]. Digital micrographs at different magnification were taken.

An imbibition test was conducted to determine the impermeability of the seed coat. Four repetitions with 25 seeds each were done. First, dried seeds were weighed and then placed in Petri dishes with Whatman filter grade 1 immersed in distilled water. To calculate the increase in fresh weight (%Wr), the seeds were weighed at the following times: 0.5, 1.5, 3.0, 6.0, 9.0, 12.0, 24.0 and 36.0 h. For each measurement, excess water was removed in order to avoid the influence of the water. The fresh weight (% Wr) was calculated with the formula % Wr = [(Wf − Wi)/Wi] × 100, where Wi is the initial weight of the seeds and Wf is the weight of the seeds over time [13].

### Evaluation of seed viability

To estimate the percentage of seed viability, a tetrazolium test was conducted on two sets of seeds collected in 2016 and 2017. The seeds were submerged in distilled water at room temperature (23°C) for 24 h in order to initiate the process of imbibition. Afterwards, the seeds were cut transversely and submerged in a solution of 2,3,5-triphenyl-tetrazolium chloride (Golden Bell Reagents^®^). Seven runs were done in which we evaluated two concentrations of the tetrazolium solution (0.5 and 1%) and three exposure times (1, 2, and 3 h) at 40°C, following the recommendations of the International Rules for Seed Testing [14]. Replicas of 10 seeds were conducted for each of the treatments. The evaluation of viability was done qualitatively by staining intensity. Embryos that stained 100% carmine red were considered viable, whereas those that stained irregularly (less than 100%) and with red or milky white tones were considered not viable.

### Evaluation of the germination of seeds of *T*. *diffusa*

#### Pre-germination treatment: Ant nest conditions

In order to simulate the seed-ant interaction through both the consumption of the elaiosome as well as the environmental conditions within an ant nest, the following treatment was done: a set of 360 seeds (L1) had the elaiosome manually removed. The seeds were then maintained in a Prendo^®^ CBRF-20 growth chamber with environmental conditions of 18°C, 70% relative humidity (RH), and 24 h of darkness (simulating environmental ant nest conditions). To avoid fungal growth during the simulation of conditions within the ant nest, the experiment was conducted for 15 days.

With the purpose of simulating the seed-ant nest interaction through the consumption of the elaiosome but without actual placement in an ant nest, the following treatment was done: a set of 360 seeds (L2) had the elaiosome manually removed, and the seeds were maintained at 23°C, 45% RH, and a photoperiod of 12/12 h (simulating environmental conditions of the study site), during a period of 15 days.

As a control, a third lot of 360 seeds (L3) was conserved without removing the elaiosome or simulating the conditions of an ant nest. The set was kept at environmental conditions of 23°C, 45% RH, and a photoperiod of 12/12 h, during a period of 15 days.

#### The effect of GA_3_ on breaking seed dormancy

Once 15 days had passed since the pre-germination treatments, germination of the three lots of seeds (Lot 1, Lot 2, and Lot 3) was evaluated under the same environmental conditions using a Prendo^®,^ CBRF-20 refrigerated germination chamber set at 28°C, 30% RH, and a 12/12 h photoperiod. The environmental conditions were maintained constant for the three seed lots during the entire experiment, but the concentration of GA_3_ was adjusted, using as a reference the study of physiological dormancy in Damiana by Viesca [15]. Two concentrations of GA_3_ were evaluated, 300 ppm and 500 ppm, in addition to a control which was not treated with GA_3_. In total, there were nine treatments with six replicas of 20 seeds each (Table 1).

**Table 1:** Pre-germination treatments and germination of seeds of *T*. *diffusa*.

	Pre-germination treatment		Germination treatment
#	A	B	C
Lot (360 seeds each)	manual removal of the elaiosome	simulating conditions within an ant nest	
**Lot 1**	Yes	Yes	Control
	Yes	Yes	300
	Yes	Yes	500
**Lot 2**	Yes	No	Control
	Yes	No	300
	Yes	No	500
**Lot 3**	No	No	Control
	No	No	300
	No	No	500

A) Pre-germination treatment by manual removal of the elaiosome; B) Pre germination treatment by simulating conditions within ant nest; C) Germination treatment 28°C, 30% RH and 12/12 h photoperiod, gibberellins (ppm).

For the treatments with GA_3_, the seeds were submerged in the corresponding concentrations for 24 h. All the seeds, including the control, were then placed in Petri dishes with a cotton pad and saturated with distilled water for 40 days. Germination was evaluated daily until day 40 of the experiment. The seeds were considered germinated if a portion of the radicle was exposed. Finally, because the seeds became infected with fungi at around 20 days, starting in the area around the elaiosome, seeds were disinfected with the fungicide CAPTAN^®^ Ultra 50 WP. A quantity of 2.25 g/L was added to the water during the final 20 days of the experiment.

#### Statistical analysis

To evaluate variability, the differences among the variables of characterization of the mature and immature seeds and the differences among the years in storage in the tetrazolium tests were determined using a Student’s T-Test. The differences among the different concentrations and times of exposure to tetrazolium were determined by an ANOVA test using the software JMP® version 7.0.

Differences in the percentage of final germination among the three pre-germination treatments (Lot 1, Lot 2, and Lot 3) and the concentrations of GA_3_ (control, 300 ppm, and 500 ppm) were determined using a Generalized Linear Model (GLM) performed with a binomial distributional error and a logit link function. When significant results were obtained, pairwise comparisons were done among the treatments.

#### Ethics statement

The thesis project from which the results of this article were obtained, was reviewed and accepted by the bioethics committee of the Faculty of Natural Sciences of the Autonomous University of Querétaro, with approval code: 88FCN2017

This research included collection of seeds, for which we obtained a field permission number 22/N1-0002/06/18 from SEMARNAT (Secretaría del Medio Ambiente y Recursos Naturales). SEMARNAT is the government ministry for the care of natural resources in Mexico.

## Results and discussion

### Morphological characterization

**Fig 1:**
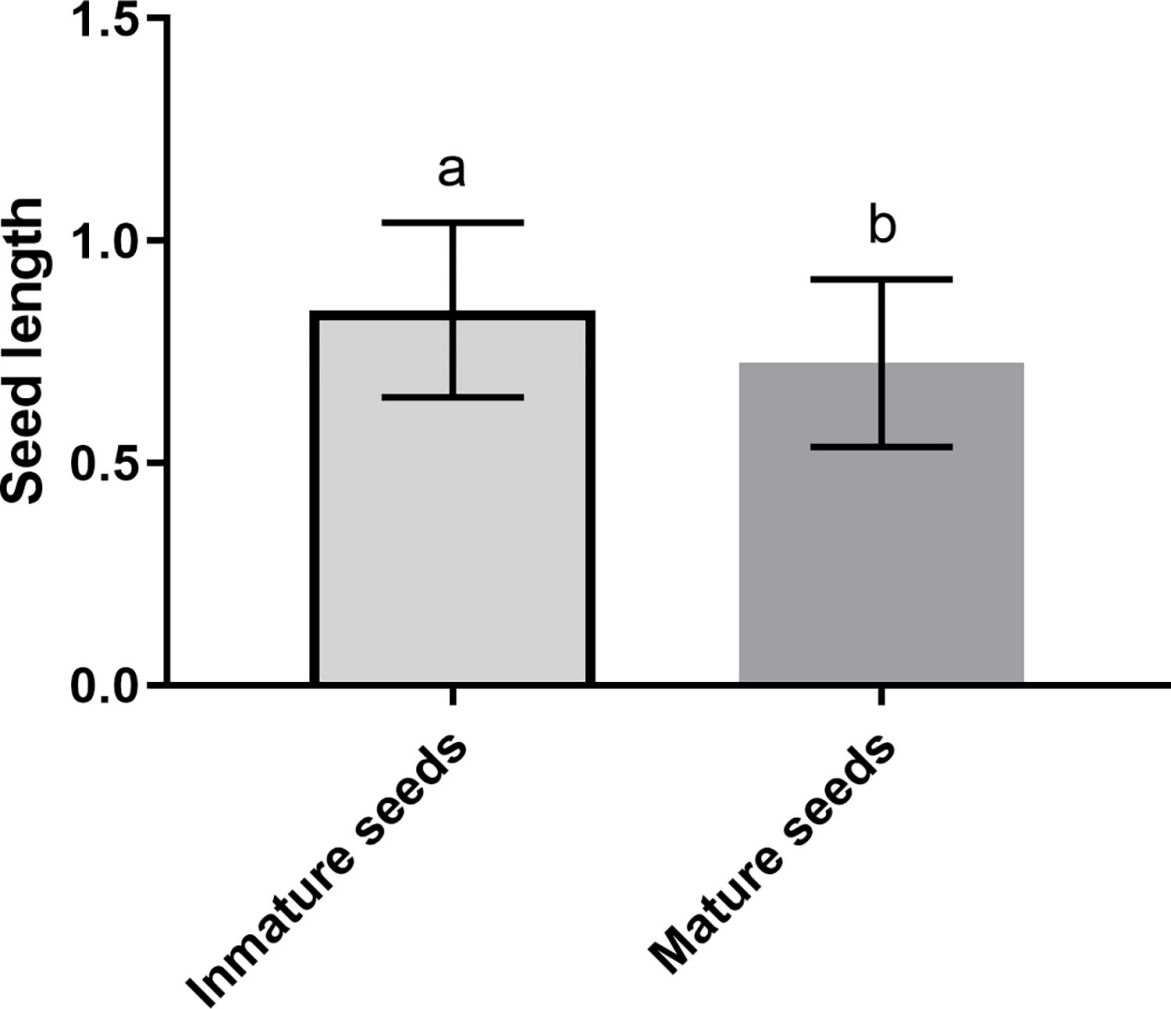
Results of the measurements of mature and immature seeds of *T*. *diffusa*. A) seed length; B) seed width (p = 0.019).

The mature seeds of *T*. *diffusa* have an average length of 0.725 mm and an average width of 0.182 mm, whereas the immature seeds have an average length of 0.840 mm and an average width of 0.152 mm. There are differences between the width of mature and immature seeds (p = 0.019, t = -2.411, d.f. = 57.884). However, there are no differences in seed length (p = 0.5801, t = 0.556, d.f. = 52.920) (Fig 1). The weight of 100 mature seeds is 0.152 g, resulting in an average seed weight of 0.0015 g.

**Fig 2:**
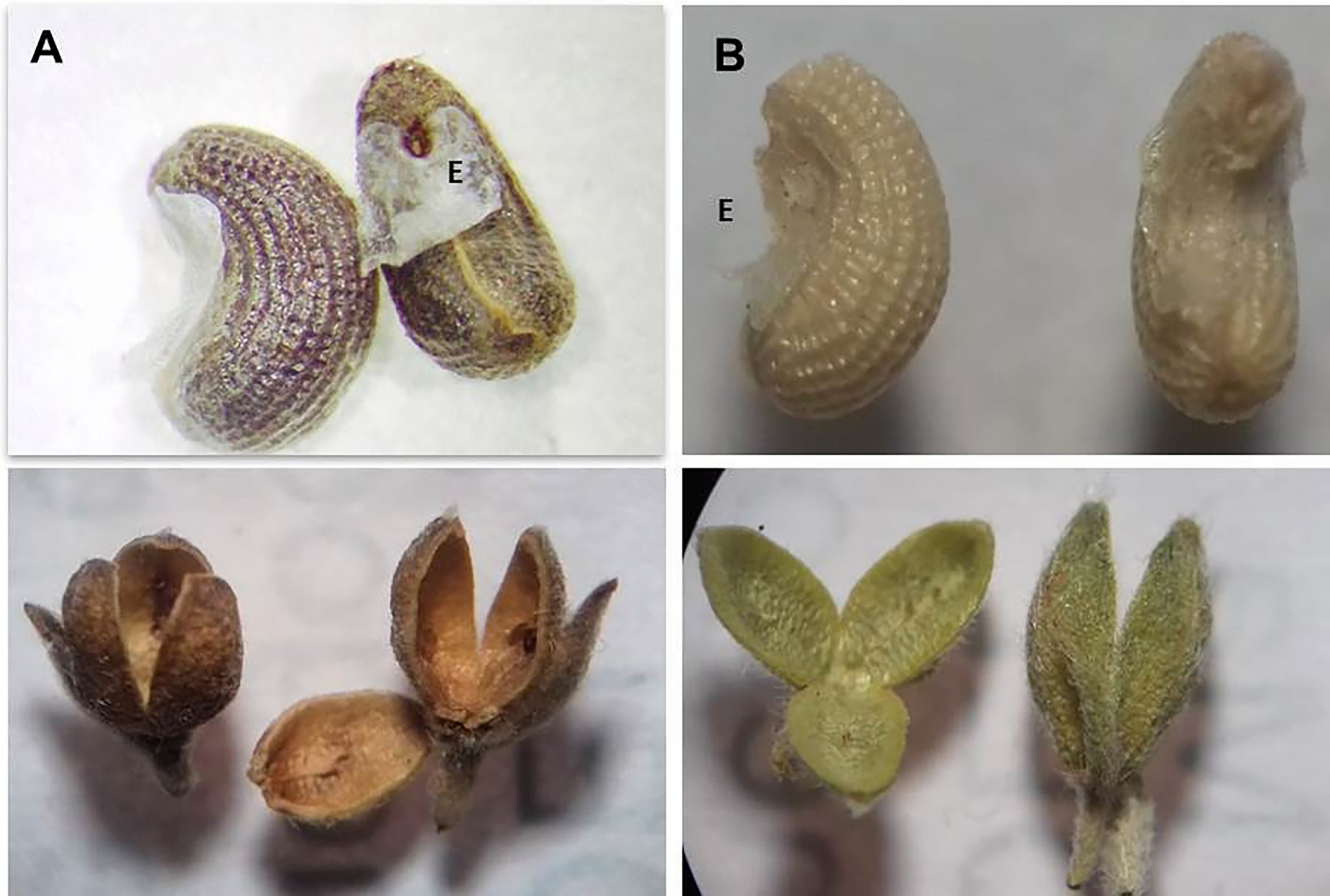
Morphological characteristics of the seeds and fruits of *T*. *diffusa*. A) Coloration and shape of the mature seeds; B) Coloration and shape of the immature seeds; C) Mature, trivalvated capsules; D) Immature, trivalvated capsules; E) Elaiosome on the mature and immature seeds. A & B 35X, C & D 20X magnification under Leica^®^ S9i dissection microscope.

**Fig 3:**
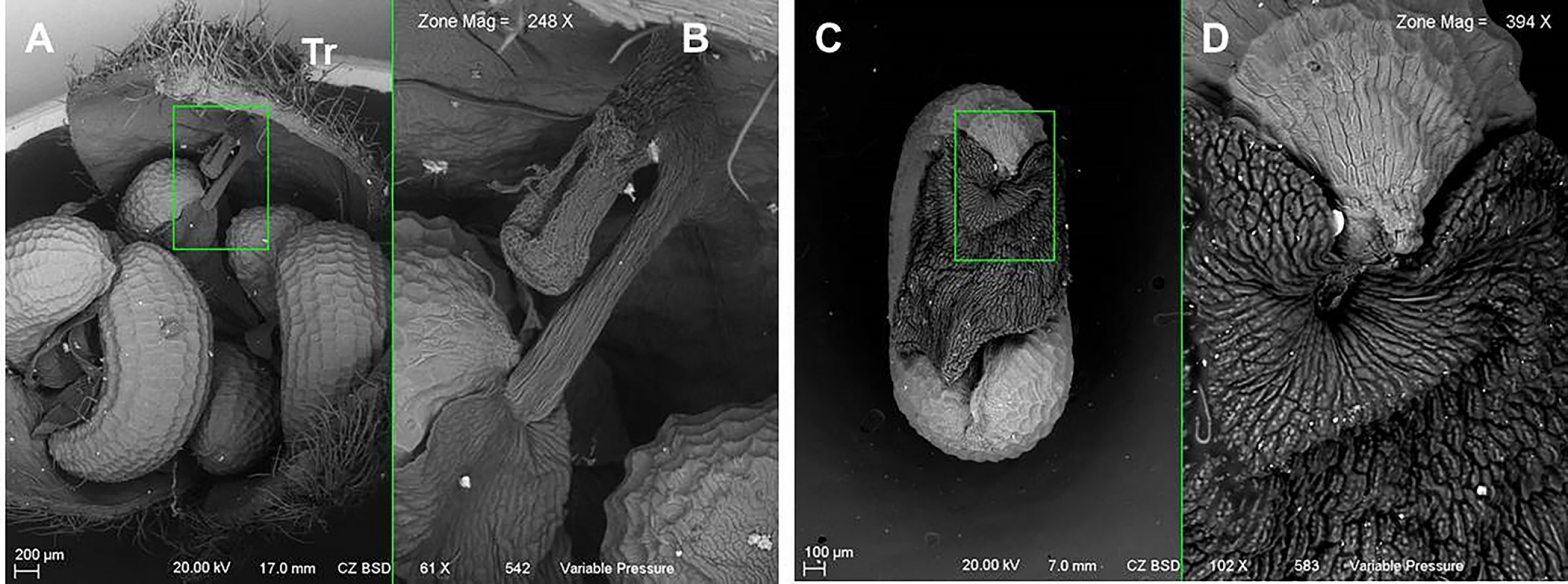
SEM images of seeds of *T*. *diffusa*. A) Capsule with seeds; B) Elaiosome insertion on the capsule, Tr = trichomes; C) *T*. *diffusa* seed; D) Elaiosome close up on seed top.

The seeds of *T*. *diffusa* turn dark brown at maturity (Munsell 7.5YR 4/2 to 7.5YR 5/2), whereas the immature seeds are whitish yellow (Munsell 2.5Y 8/4 to 2.5Y 8/6) (Table 2). The shape of both mature and immature seeds is curved, and the seed coat is reticulate and hard. The fruits are trivalvated capsules covered by trichomes and containing six seeds. These are green when young (Munsell 5Y 6/4 to 5Y 7/4) and become brown at maturity (Munsell 5Y 6/4 to 5Y 7/4) (Fig 2). The SEM images show that both mature and immature seeds are partially covered by a tissue termed an elaiosome (Fig 3).

**Table 2:** Morphological characteristics of mature and immature seeds of *T*. *diffusa*. s.e: standar error.

Morphological characteristic	Mature seeds	Immature seeds
**Weight (g)**	0.152	0.152
**Length (mm ± s.e.)**	0.725 ± 0.034	0.84 ± 0.035
**Width (mm ± s.e.)**	0.182 ± 0.031	0.152 ± 0.043
**Shape**	curved (pear-shaped)	curved (pear-shaped)
**Color**	dark brown (Munsell 7.5YR 4/2 to 7.5YR 5/2)	yellowish white (Munsell 2.5Y 8/4 to 2.5Y 8/6)

**Fig 4:**
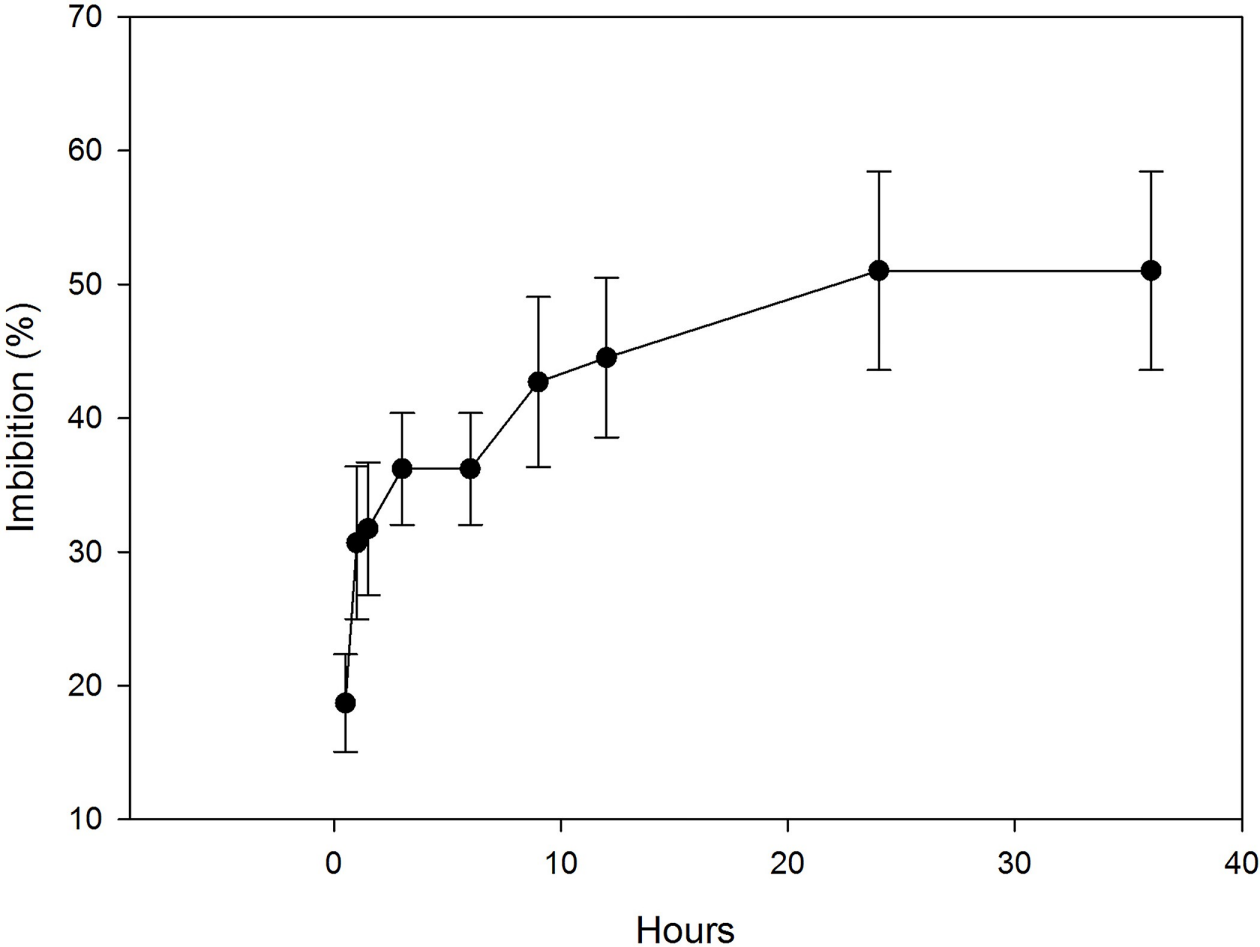
*T*. *diffusa* seeds imbibition curve. *T*. *diffusa* seeds reached a maximum absorption of 51.02% in 24 hours. Bars represent standard deviation.

During the permeability test, seeds of *T*. *diffusa* gain weight due to the absorption of water. After 0.5 h, the weight had increased 18.7 ± 5.71%, in 1 h 30.67 ± 3.64%, and after 6 h 36.20 ± 4.18%. The capacity to absorb water stabilizes after 24 h, reaching a maximum percentage of absorption of 51.02 ± 7.43% (Fig 4).

The seed size of *T*. *diffusa* agrees with that described by González and Arbo [16] and can be considered a diagnostic morphological character of the species. With regard to weight, this feature is described for the first time and represents important information for subsequent agronomic management. Seed color can be considered as agronomical diagnostic because it is markedly different between mature and immature seeds. Although there are articles on the seeds of *Turnera* [16], in this study characteristics of the shape and surface cover are described for the first time constituting an advance in our knowledge of seed features that will benefit the agronomic management of *T*. *diffusa*.

The SEM micrographs (Fig 3) show the fleshy appendages that are common in the genus *Turnera*. These were referred to as arils by [16]. However, their presence had not been specifically reported for *T*. *diffusa*. Due to them being a tissue that contains reserves of proteins, oils and starches [10, 17], it is possible to classify them more specifically as elaiosomes. The presence of elaiosomes in the genus *Turnera* has previously been documented only for *T*. *ulmifolia* and *T*. *subulata* [16], and the results of our research allow *T*. *diffusa* to be included among the species of the genus *Turnera* that possess an elaiosome.

The results of the permeability test demonstrate that seeds can absorb up to 51.02% of their weight in water, showing them to be permeable and eliminating the possibility of physical dormancy.

### Evaluation of seed viability

**Fig 5:**
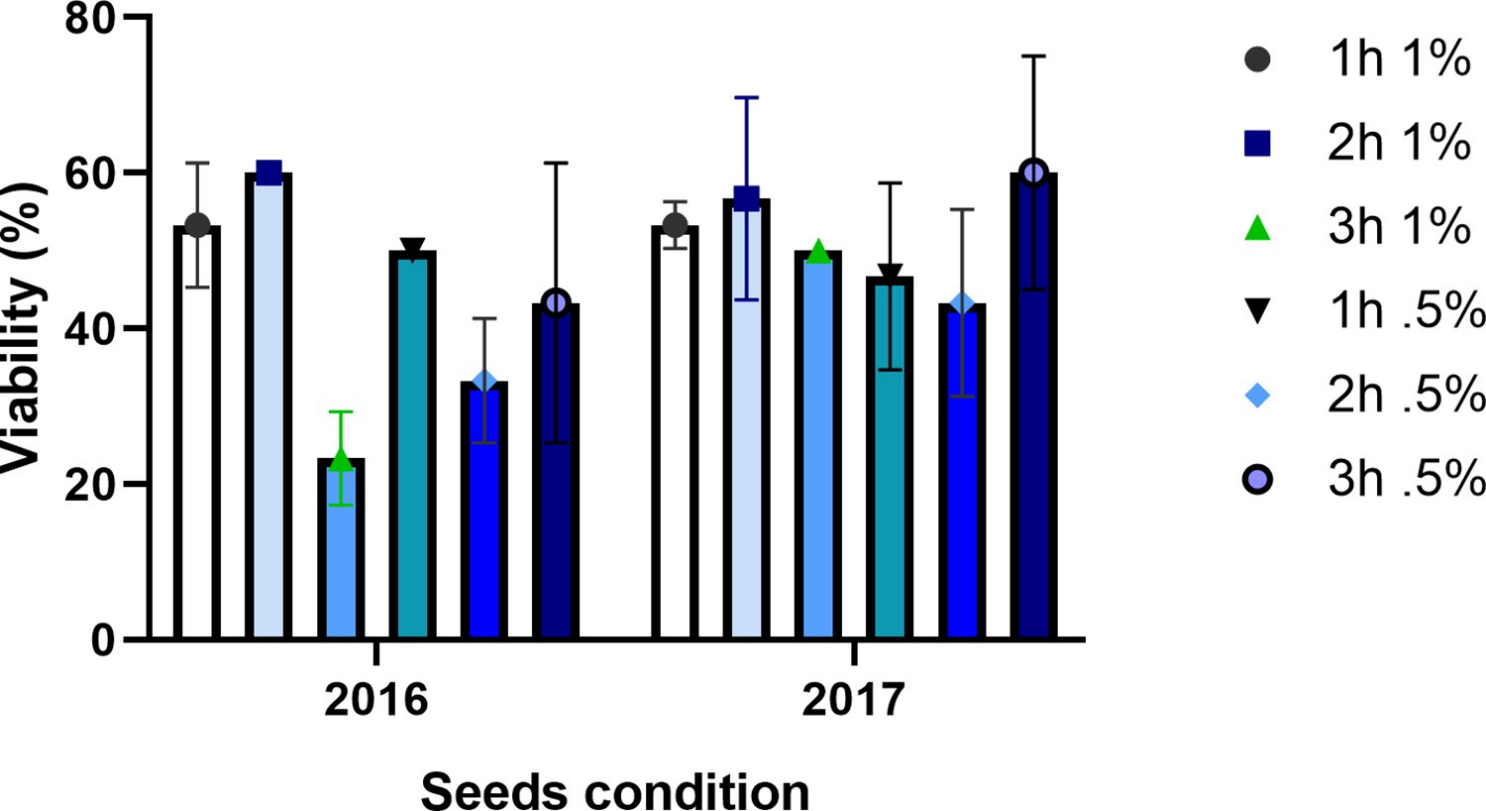
Seed viability. Percentage of staining of the seeds of *T*. *diffusa* collected in 2016 and 2017 (t = 0.200, d.f. = 33.606. p<0.05).

The seed permeability test with tetrazolium revealed no differences between the staining percentage of seeds of *T*. *diffusa* collected in the years 2016 and 2017 (p = 0.200, t = 1.304, d.f. = 33.606). Neither were there observable differences in the staining percentages in relation to the tetrazolium concentration and the time of exposure to the solution (F_5, 30_ = 1.449, p = 0.235). For seeds collected in 2016, the highest staining percentage was 60% under the following conditions: 1% concentration of tetrazolium solution for 2 h at 40°C. The lowest staining percentage was 23% under the following conditions: 1% concentration of tetrazolium solution for 3 h at 40°C (Fig 5). For seeds collected in 2017, the highest staining percentage was also 60% using the following conditions: 0.5% concentration of tetrazolium solution for 3 h at 40°C. The lowest staining percentage was 40% under the following conditions: 0.5% concentration of tetrazolium solution for 2 h at 40°C. Seed viability of *T*. *diffusa* was not affected by storage time over the year since both sets reached a staining percentage of 60% (Fig 5).

In this study, no significant differences in seed viability were determined for the collections made a year apart. Although previous research has documented that seeds lose their viability over the year [18], this effect was not obvious in seeds stored for a single year. To the contrary, the results of the tetrazolium test showed that seeds collected in 2016 (exposed to 1% concentration of tetrazolium for 2 hours) had a staining percentage of 60%, whereas the seeds from 2017 exposed to the same conditions, had a 56% staining percentage. In other words, younger seeds stained less. These results showing a lower staining percentage could indicate that at the time of the tetrazolium test, the embryos of *T*. *diffusa* were physiologically immature, an aspect related to a decrease in enzymatic activity and as a consequence a decrease in staining percentage. The results of the viability test agree with those of the germination test, indicating that the seeds have physiological dormancy. From an ecological viewpoint, the physiologically immature embryos of seeds collected within the year indicate that the species forms a seed bank and that the seeds probably germinate the year after being produced by the plant.

### Evaluation of seed germination of *T*. *diffusa*

The percentage of germination of *T*. *diffusa* was significantly different depending on the pre-germination treatment (X^2^ = 55.131, d.f. = 2, p = < .0001), and the pairwise statistical analysis showed significant differences among all the pre-germination treatments analyzed. With regard to the germination treatments, no significant differences were detected between the gibberellin concentrations of 300 ppm and 500 ppm (X^2^ = 20.453, d.f. = 2, p = < .0001), but there were significant differences between seeds treated with gibberellins and the control (Table 3).

**Fig 6:**
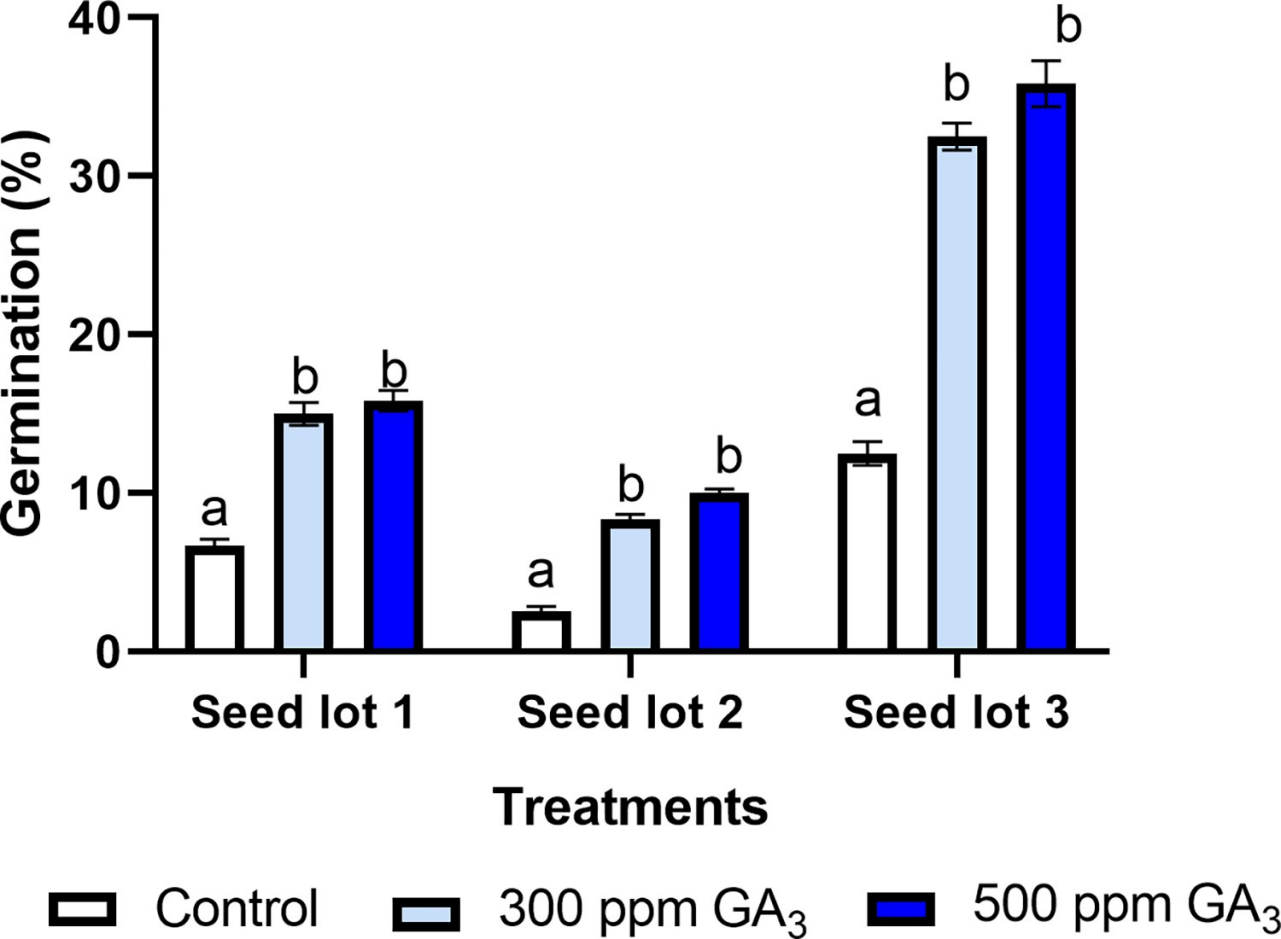
Percentage of seed germination of *T*. *diffusa* under two distinct pre-germination treatments: Simulation of conditions within an ant nest (18°C, 70% RH, 24 h darkness) and room temperature, with two concentrations of GA_3_, 300, 500 and a control without application of GA_3_. Lot 1, conditions of an ant nest and without the elaiosome. Lot 2, without conditions of an ant nest and with the elaiosome. Lot 3, without conditions of an ant nest and without the elaiosome.

Lot 1. The treatment simulating conditions within an ant nest and with the removal of the elaiosome achieved the following maximum percentages of germination: 16% using 500 ppm of GA_3_, 15% using 300 ppm of GA_3_, and 2.5% without the application of GA_3_ (Fig 6).

Lot 2. The treatment without the conditions within an ant nest and with the removal of the elaiosome achieved the following maximum percentages of germination: 10% using 500 ppm of GA_3_, 8% using 300 ppm of GA_3_, and 1.66% without the application of GA_3_ (Fig 6).

Lot 3. The treatment without the conditions within an ant nest and with the presence of the elaiosome achieved the following maximum percentages of germination: 36% using 500 ppm of GA_3_, 32% using 300 ppm of GA_3_, and 12.5% without the application of AG_3_ (Fig 6).

**Table 3:** Pairwise comparison of the germination percentage for the pre-germination treatments and GA_3_ concentrations *p* <0.05. Means with the same letter are not significantly different (*p* ≤ 0.05).

GA_3_	Seed lot 1	Seed lot 2	Seed lot 3	Average
**0**	6.7 ± 5.2 c	2.5 ± 4.2 c	12.5 ± 9.4 c	7.2 ± 7.5 b
**300**	15 ± 8.9 c	8.3 ± 4.1 c	32.5 ± 10.4 ab	18.6 ± 13.0 a
**500**	15.8 ± 8.0 bc	10.0 ± 3.2 c	35.8 ± 17.7 a	20.6 ± 15.6 a
**Average**	12.5 ± 8.3 b	6.9 ± 4.9 b	26.9 ± 16.2 a	

Seed germination of *T*. *diffusa* can be considered irregular since it varies between six and 39 days. In most of the treatments, germination ceased at day 31, but in one treatment, germination continued until day 39.

The three pre-germination treatments conducted here resulted in low percentages of germination. The highest percentages were 36% and 31%, at concentrations of 500 and 300 ppm of GA_3_, respectively. However, in comparison with the studies conducted by Viesca [15], in which 25% germination was the maximum obtained, the results of our study show the highest germination percentages obtained to date. None of the previous studies on the germination of *T*. *diffusa* had employed pre-germination treatments based on ant-seed interactions.

The pre-germination treatment imitating the environmental conditions of an ant nest and the removal of the elaiosome do not promote an increase in the percentage of seed germination in *T*. *diffusa*. These results are similar to those of Cuautle [10] and Rocha [19] for *T*. *ulmifolia* and *T*. *subulata*, in which the species of ants studied were attracted by the elaiosome to consume it, but none of the associated ants actually favored an increase in the germination success of the seeds. The results of our study under laboratory conditions show that the removal of the elaiosome is not a factor that benefits germination, although it should be considered as a mechanism to promote its dispersion by ants.

Concerning germination treatments, the addition of GA_3_ increased the percentage of germination, and these results indicate that *T*. *diffusa* has primary dormancy. Morphological primary dormancy is ruled out because the results from the imbibition test show that seeds absorb up to 51.02% of their weight in water during a period of 24 hours. The species is proposed to have primary physiological dormancy due to the results of the germination treatments coupled with the results of the viability test, suggesting immaturity of the embryo at the moment of germination. Although in the present study we did not obtain high percentages of germination, our results are relevant because they provide the basis for future research focusing on breaking this type of dormancy.

With regard to the presence of the elaiosome on the seeds, the results of this study indicate that its primary function relates to seed dispersal. From the standpoint of seed handling for the propagation of the species, it is necessary to apply treatments to inhibit fungal growth, since the high content of nutrients contained in the elaiosome favor fungal proliferation. Rocha [19] proposes that during the consumption of the elaiosome, the ants introduce some type of salivary substance that promotes scarification and optimizes germination. However, in the case of *T*. *diffusa*, removal of the elaiosome is shown to not benefit germination.

In order to obtain better information about propagation of *T*. *diffusa*, it is necessary to study effective pollination, colonization capacity of the species *in situ*, fecundity, and the reproductive rate of the populations. At present, the efficient method for propagation of *T*. *diffusa* is by cuttings, but propagation by seeds has various advantages. These include maintaining the genetic diversity of the species in terms of production, genetic improvement, domestication, and conservation by sexual propagation.

## Conclusions

The characterization of *T*. *diffusa* seeds represents an important contribution to our agronomic understanding of the species. It also shows the presence of an elaiosome on the seeds, a characteristic associated with harvester ant interactions. The seeds absorb 51.02% of their weight in water during the first 24 hours of imbibition, for which we can rule out morphological dormancy. The results obtained in the viability test allow the distinction between viable and non-viable seeds, with the optimal conditions for this test being a treatment with 1% tetrazolium for two hours at 40°C. The pre-germination treatment of removing the elaiosome and imitating conditions within an ant nest did not result in an increase of germination percentage. The germination treatment with GA_3_ increased the germination percentage, which leads to the conclusion that the species has primary physiological dormancy.
